# Genome‐wide analysis of somatic noncoding mutation patterns and mitochondrial heteroplasmic shift in type B1 and B2 thymomas

**DOI:** 10.1002/path.6496

**Published:** 2025-12-04

**Authors:** Kohei Fujikura, Isabel Correa, Susanne Heck, Kaoru Watanabe, Juliet King, Emma McLean, Susan Ndagire, Yoshihisa Takahashi, Masahiko Kuroda, Andrea Bille, Daisuke Nonaka

**Affiliations:** ^1^ Department of Diagnostic Pathology, Graduate School of Medicine Kobe University Kobe Japan; ^2^ Medical School Building, St Mary's Campus, Norfolk Place Imperial College London London UK; ^3^ Institute of Diabetes Research, Helmholtz Zentrum Munchen Munich Germany; ^4^ Department of Computer Science Osaka Electro‐Communication University Osaka Japan; ^5^ Department of Thoracic Surgery Guy's and St Thomas’ NHS foundation trust London UK; ^6^ Department of Cellular Pathology Guy's and St Thomas’ NHS foundation trust London UK; ^7^ King's Health Partners Cancer Biobank Cancer Centre at Guy's, Guy's and St Thomas’ NHS Foundation Trust London UK; ^8^ Department of Molecular Pathology, Graduate School of Medicine Tokyo Medical University Tokyo Japan; ^9^ Cancer Centre, School of Cancer and Pharmaceutical Sciences King's College London London UK; ^10^ Department of Histopathology Guy's and St Thomas’ NHS foundation trust London UK; ^11^ Department of Cellular Pathology King's College London London UK

**Keywords:** thymoma, whole genome sequencing, DEPArray, noncoding mutation, mitochondrial heteroplasmy, cis‐regulatory element, mutational signature

## Abstract

Type B1 and B2 thymomas are lymphocyte‐rich malignant tumours with few somatic mutations in protein‐coding regions of the nuclear genome; nonetheless, noncoding regions remain uncharacterized. Here, we developed a method to isolate pure thymoma cells from lymphocyte‐rich tissues, and then performed genome‐wide deep sequencing. The total number of somatic mutations was ~80 times higher in noncoding regions than in coding regions in type B12 thymomas (1,671.3 versus 21.1 per case). Coding mutations were identified in epigenetic regulators, DNA repair genes, and some other genes. Nevertheless, 40% of the cases exhibited fewer than four nonsynonymous mutations in coding regions. A systematic noncoding analysis identified 405.0 mutations per case in cis‐regulatory elements and detected six recurrent mutations: one interferon regulatory factor (*IRF8*), two E3 ubiquitin ligases (*UBR2* and *RNF213*), and three intergenic regions. Tumour‐specific/enriched mitochondrial heteroplasmic shift was observed in 90% of cases, with a significant proportion of mutations located in the D‐loop region. When tracing the evolutionary lineage of mtDNA mutation, the majority of cases can be explained by a linear evolutionary model. This suggests that positive selection may be operating on the mitochondrial genome during thymoma development. In summary, numerous noncoding mutations and mitochondrial heteroplasmic shift were detected in type B1 and B2 thymomas, some of which may be functional. Given the paucity of coding mutations observed in this disease entity, other factors such as disruption of the noncoding landscape and tumour‐specific/enriched mitochondrial heteroplasmic shift, may contribute to the development of thymoma. © 2025 The Author(s). *The Journal of Pathology* published by John Wiley & Sons Ltd on behalf of The Pathological Society of Great Britain and Ireland.

## Introduction

Thymomas are a type of thymic epithelial neoplasm that exhibit organotypical features observed in either the active or the senescent thymic gland [1]. According to the World Health Organization (WHO) classification, they are divided into type A, B, and AB (WHO Classification of Tumours Editorial Board). Thoracic Tumours. Lyon (France): International Agency for Research on Cancer; 2021. (WHO Classification of Tumours, 5th edition, vol. 5) [2, 3]. Type B is further subdivided into types B1, B2, and B3 based on the relative proportion of the nontumourous lymphocytic component, and the resemblance to normal thymic architecture [4, 5]. It has been demonstrated that different histologic subtypes of thymomas have distinct prognoses and clinical features [6, 7]. For instance, type B thymomas have been found to have a higher stage (20%–50% in stage III–IV) than type A and AB thymomas (90% in stages I–II) [6]. In addition, a greater incidence of autoimmune diseases has been observed in patients with type B thymoma than in patients with type A thymoma [7]. Surgery is the standard treatment for localised tumours, with postoperative radiotherapy and chemotherapy reserved for advanced stages [8]. However, effective postoperative treatment has not been developed, possibly due to a lack of basic and clinical research compared to other malignancies.

Recent genomic studies of thymic epithelial tumours have revealed that type A and AB thymomas are characterized by a high frequency of thymoma‐specific codon mutations (L424H) in the *GTF2I* gene [9, 10]. On the other hand, no recurrent genetic abnormalities have yet been identified in the type B group, although a *KMT2A*::*MAML2* gene fusion has been reported in a small subset of type B2‐3 thymomas [11].

It has been suggested that B1 and B2 thymomas exhibit a markedly reduced number of somatic mutations in the protein‐coding regions of the nuclear genome in comparison to other malignancies [10, 12]. Thus, it is plausible that mutations in noncoding regions play a pivotal role in the pathogenesis of thymomas, as has been demonstrated in certain cancer types [13, 14, 15, 16]. Nevertheless, the noncoding regions of thymomas remain uncharacterized. In addition, type B1 and B2 thymomas contain a significant proportion (50%–95%) of nonneoplastic immature T lymphocytes. The presence of a large nonneoplastic component renders genetic analysis of thymomas exceedingly challenging. In order to overcome these challenges and carry out genome‐wide analysis, we developed a method for isolating pure tumour cells from frozen tissue sections. Subsequently, we conducted deep whole‐genome sequencing and employed computational methods to identify significantly mutated noncoding regions and mitochondrial DNA.

## Materials and methods

Additional details are provided in the Supplementary materials and methods (available online).

### Ethics approval and consent

The study was approved by the Research Ethics Committee of King's Health Partners Cancer Biobank (KHP Cancer Biobank REC reference 18/EE/0025). All procedures conducted in the study were in accordance with the 1964 Declaration of Helsinki [17] and its later amendments or comparable ethical standards. Written informed consent for research was provided by all patients before surgical treatment was initiated.

### Patients

Type B1 and B2 thymomas were retrieved from the King's Health Partners Cancer Biobank. All tumours were treated by surgical resection for curative intent, without prior adjuvant therapy. The diagnosis was confirmed by reviewing haematoxylin and eosin (H&E)‐stained sections from resected tumours in accordance with the 2021 World Health Organization classification of thymic tumours [2, 3]. The clinicopathologic information of the patients is presented in the supplementary material, Table S1. The median age of the patients at the time of surgery was 51 years (range, 29–79 years), with seven males and three females. Three patients exhibited type B1 thymomas, while seven exhibited type B2 thymomas. Six patients presented with paraneoplastic disease, including five with myasthenia gravis and one with autoimmune hepatitis. At the time of diagnosis, eight patients (80%) were staged as Masaoka stage I–II, while two (20%) were stage III–IV. The median tumour size was 60 mm. Five patients (50%) had a positive margin (R1) and received postoperative radiotherapy for their primary tumours. One patient experienced recurrence. All patients demonstrated no evidence of other malignancies.

### Cell isolation from frozen tissue

Frozen thymoma sections (60 μm thickness) were fixed in 2% paraformaldehyde at room temperature for 15 min in an Eppendorf tube. The sections were then washed four times with 1 ml of PBS and centrifuged at 800× g for 5 min. The sections were then digested in a solution of a mixture of 0.1% collagenase (Sigma‐Aldrich, St. Louis, MO, USA) and 0.1% dispase (Gibco, Grand Island, NY, USA) at 37 °C for 15–30 min. Subsequently, 5 ml of RPMI 1640 (Gibco) with 10% foetal calf serum (Gibco) was added together with EDTA to a final concentration of 2 mm. The samples were then centrifuged at 460× g for 10 min and resuspended in 1 ml of FACS buffer (1% BSA and 2 mm EDTA in PBS). The cells were counted, and 1–2 × 10^5^ cells were incubated with Fc block (Biolegend, San Diego, CA, USA), followed by APC anti‐human CD45 (clone HI30; BioLegend), PE anti‐human CD205 (clone HD30; BioLegend) antibodies, and Hoechst (Thermo Fisher Scientific, Waltham, MA, USA).

The isolation of tumour and matched normal (used as baseline) cells was conducted using a DEPArray V.II system with A300K cartridges (Menarini Silicon Biosystems, Philadelphia, PA, USA). The CD45^+^CD205^−^ and CD45^−^CD205^+^ cell populations were used as nonneoplastic and neoplastic fractions, respectively, and these cells were isolated and sequenced separately. Approximately 10,000 cells were loaded into cartridges and the selected cells were eluted as single cells using PCR tubes for collection. The supernatant was then removed, and genomic DNA amplified using the Ampli1 WGA kit (Menarini Silicon Biosystems) following the manufacturer's protocol, after which samples were mixed in equal amounts.

### Deep whole‐genome sequencing

The whole‐genome sequencing (WGS) library was constructed using the NEBNext Ultra™ II DNA Library (New England Biolabs, Ipswich, MA, USA). The samples were subjected to sequencing using a 2 × 150 paired‐end configuration. At least 288 gigabases of raw sequencing data were generated for all samples.

### Read mapping and detection of somatic mutations

Raw sequencing data were processed using the DRAGEN DNA Pipeline on the Illumina DRAGEN Bio‐IT Platform v4.1.7 (Illumina, San Diego, CA, USA). Following adapter trimming, the sequencing reads were aligned to GRCh38/hg38. Mutations were filtered based on the following criteria: (1) ‘PASS’ in the quality filter; (2) somatic Qscore ≥18; (3) tumour sample coverage ≥ 21×; (4) normal sample coverage ≥ 21×; (5) tumour frequency > 0.25; (6) ≥ 6 distinct reads supporting the mutation in the tumour sample; and (7) normal frequency < 0.02. Both baseline and tumour data were analysed simultaneously, and variants present only in tumour cells were extracted using the variant caller. The variants in exonic regions were classified into six categories: synonymous, nonsynonymous, splice acceptor, splice donor, stop‐causing, and stop‐loss variants. All the exonic variants were visually inspected using the Integrative Genomics Viewer (IGV) (http://software.broadinstitute.org/software/igv/; accessed on 27 January 2025) to exclude potentially artefactual variants as previously described [18, 19]. These included variants occurring in variant‐rich regions or variants identified exclusively at read ends. All filtered somatic variants were annotated with Variant Effect Predictor.

### Tumour mutational burden

Tumour mutational burden (TMB) was defined as the number of somatic coding base substitutions and indel mutations per megabase of genome examined. The calculation encompasses all base substitutions and indels in the coding region, including synonymous alterations. Noncoding alterations were not included in the analysis. TMB data across other cancer types were obtained from a previous study [12]. These data also only cover mutations in the coding regions.

### Characterisation of coding mutations

The functional impact of an amino acid substitution on the protein structure and overall function was predicted using SIFT [20] and PolyPhen [21]. The gene ontology (GO) terms of molecular functions, cellular component, and biological processes of the detected mutations were searched using the web‐based tool Metascape (https://metascape.org/gp/index.html#/main/step1; accessed on 2 October 2024). The mutated genes were analysed using the STRING database (http://string-db.org; accessed on 17 October 2024) to evaluate their relationships through protein–protein interactions (PPI). The PPI data were imported into Cytoscape (https://cytoscape.org/; accessed on 30 November 2024), which was used to evaluate the relationship between mutant proteins in type B1‐2 thymoma.

### Identification of recurrent noncoding mutations

We next examined whether mutations in the noncoding regions of type B1 and B2 thymomas were located in the recurrently mutated regions identified in previous studies. A list of previously reported noncoding mutations in different cancer types was retrieved from ten international noncoding projects, including pancancer [13, 14, 22, 23, 24, 25], hepatocellular carcinoma [15], breast cancer [16], urinary bladder cancer [26], and diffuse large B‐cell lymphoma projects [27], all of which clearly defined the chromosomal positions.

*De novo* detection of recurrent mutation was performed using the MutSpot R package [28] with the default settings. Manhattan plots were generated based on the chromosomal position and *p* value. A false discovery rate (FDR) < 0.05 was defined to be significant.

### Characterisation of noncoding mutations

The ENCODE Registry [29] of candidate cis‐Regulatory Elements (cCREs) was used to identify DNA regulatory elements in the human genome. The ReMap 2022 Atlas of regulatory regions, which is an integrative analysis of all public ChIP‐seq data for transcriptional regulators from GEO, ArrayExpress, and ENCODE was retrieved from the UCSC genome browser or ReMap2022 database (https://remap.univ-amu.fr/; accessed on 12 January 2024). The dplyr R package was used to ascertain whether the identified gene mutations were located on the cis‐regulatory elements.

### Detection of copy number alterations

Sequencing reads were counted in 500 kb bins. Segmentation was conducted using Shifting Level Models with the disabled merging of two adjacent segments.

### Detection of mtDNA mutations

Raw sequencing data were processed using the DRAGEN DNA Pipeline on the Illumina DRAGEN Bio‐IT Platform v4.1.7 (Illumina). After adapter trimming, the sequencing reads were aligned to GRCh38/hg38. Subsequently, mtDNA mutations were filtered based on the following criteria: (1) ‘PASS’ in the quality filter; (2) Qscore ≥ 18; (3) tumour frequency > 0.05; (4) ≥ 5 distinct reads supporting the mutation in the tumour sample; (5) normal frequency < 0.05. Both baseline and tumour data were analysed simultaneously, and variants present only in tumour cells were extracted using the variant caller. We also attempted to reduce any contribution in variability from mitochondrial DNA sequences present in NUMTs by exclusively utilizing read‐pairs in which both reads were mapped to the mitochondrial genome.

The heteroplasmy level was defined as the proportion of mutant reads in the total reads for a given mutation site. Our analysis demonstrated that sequencing coverage had no notable effect on heteroplasmy level, thereby providing strong evidence for the high technical reliability of the method.

The mitochondrial phylogenetic tree was constructed according to the following three rules, since in the case of mitochondria with multiple copies within a single cell, their phylogenetic relationships cannot be inferred by existing methods.

1. The VAFs for each genetic variant (A1, A2, … An) are ordered from highest to lowest ratio, as shown below:

A0:100%>A1≧A2≧…≧An

2 The branches of the phylogenetic tree are extended one by one, starting from the genetic variant with the largest VAF (A1, A2, … An).
3 Ai < ∑Aj, (where j is an element of J ⊆ {i + 1, … *n*}), then we do not branch from Ai to Aj

### Analysis of scRNA‐seq thymoma data

The publicly available data for the scRNA‐seq dataset of thymoma samples were obtained from the SingleCellPortal (https://singlecell.broadinstitute.org/single_cell; accessed on 12 March 2024). The thymoma study included four primary tumour and blood samples. The blood samples and type AB thymoma samples were excluded from our study. Expression levels (transcripts per million, or TPM) were obtained, with tumour cells identified using previously determined criteria. Uniform manifold approximation and projection (UMAP) representations of the cells were generated from the total expression profiles. TPM values were then compared between the given factors.

### Statistical analyses

All statistical analyses were carried out using GraphPad Prism version 10 for Windows (GraphPad Software, San Diego, CA, USA). The Mann–Whitney *U* test or the Wilcoxon signed‐rank paired test was used to compare the difference between two groups with continuous variables. The Fisher's exact test was used for discrete variables. All *p* values were calculated using the two‐tailed test, and statistical significance was set at the level of *p* < 0.05 or FDR < 0.05.

## Results

### Strategy for analysing type B1 and B2 thymoma genome

**Figure 1:**
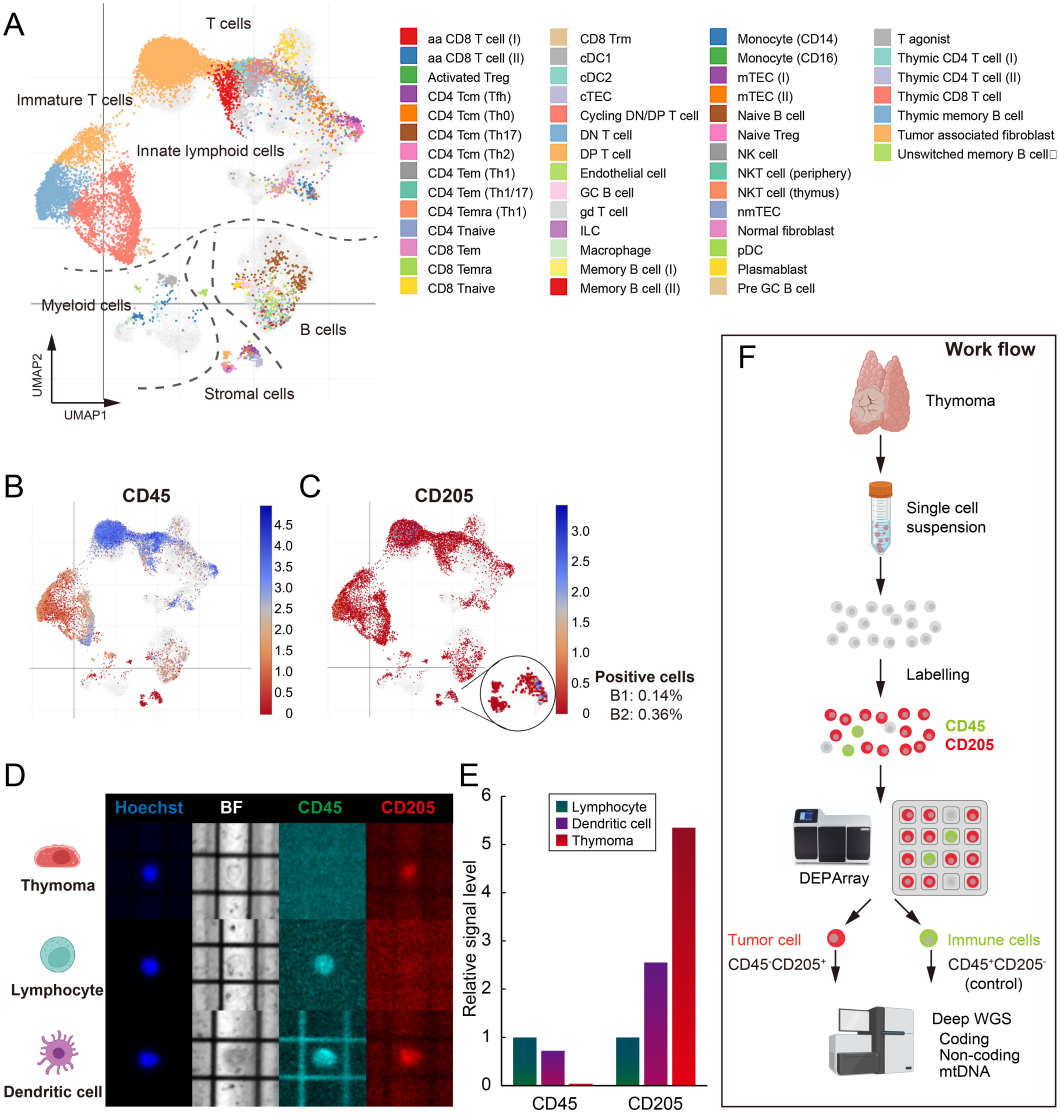
Strategy for isolating pure tumour cells. (A) Uniform manifold approximation and production (UMAP) plot displaying 49 clusters from thymoma patients (total 15,731 cells). (B and C) UMAP plot of marker CD45 (PTPRC) and CD205 (LY75). (D) Representative images of cells on the DEPArray. Cells were stained with CD45, CD205, and Hoechst for identification and selection. (E) Quantification of signal levels of CD45 and CD205 in each cell type. (F) Workflow to rigorously isolate thymoma and matched control cells for deep whole‐genome sequencing. This figure was created using BioRender (https://BioRender.com).

To comprehensively detect genome‐wide mutations in nonneoplastic lymphocyte‐rich tissues, we aimed to develop a rigorous tumour isolation method that preserves genomic quality and reduces various biases. First, we analysed publicly available data on scRNA‐seq datasets of thymoma samples to determine the specificity of existing tumour‐specific markers in type B1 and B2 thymomas. The scRNA‐seq data demonstrated that thymoma tissue is composed of large T‐cell, B‐cell, and myeloid cell clusters, and a very small number of stromal cells, including tumour cells (Figure 1A). CD205 (DEC205/LY75) is a specific surface marker for thymoma that we have proposed (supplementary material, Figure S1A) [30], when combined with CD45, it is possible to strictly isolate a small number of tumour cells (Figure 1B,C; supplementary material, Figure S1B). The theoretical percentage of CD45^−^CD205^+^ tumour cells was 0.14% for B1 thymoma and 0.36% for B2 thymoma in the scRNA‐seq dataset. The CD45^+^CD205^−^ cell population (> 95%) was considered nontumourigenic and best suited as a matched control for whole‐genome sequencing. The CD45^+^CD205^+^ cell population was small and mostly dendritic cells. These results raise the question whether some existing genetic analyses have been performed with sufficient tumour cellularity, and demonstrate the importance of our research strategy. Here, we used the DEPArray image sorter to isolate pure tumour cells (Figure 1D,E), and extracted genomic DNA for genome‐wide identification of somatic alterations (Figure 1F).

### Characterisation of type B1‐2 thymoma genomes

**Figure 2:**
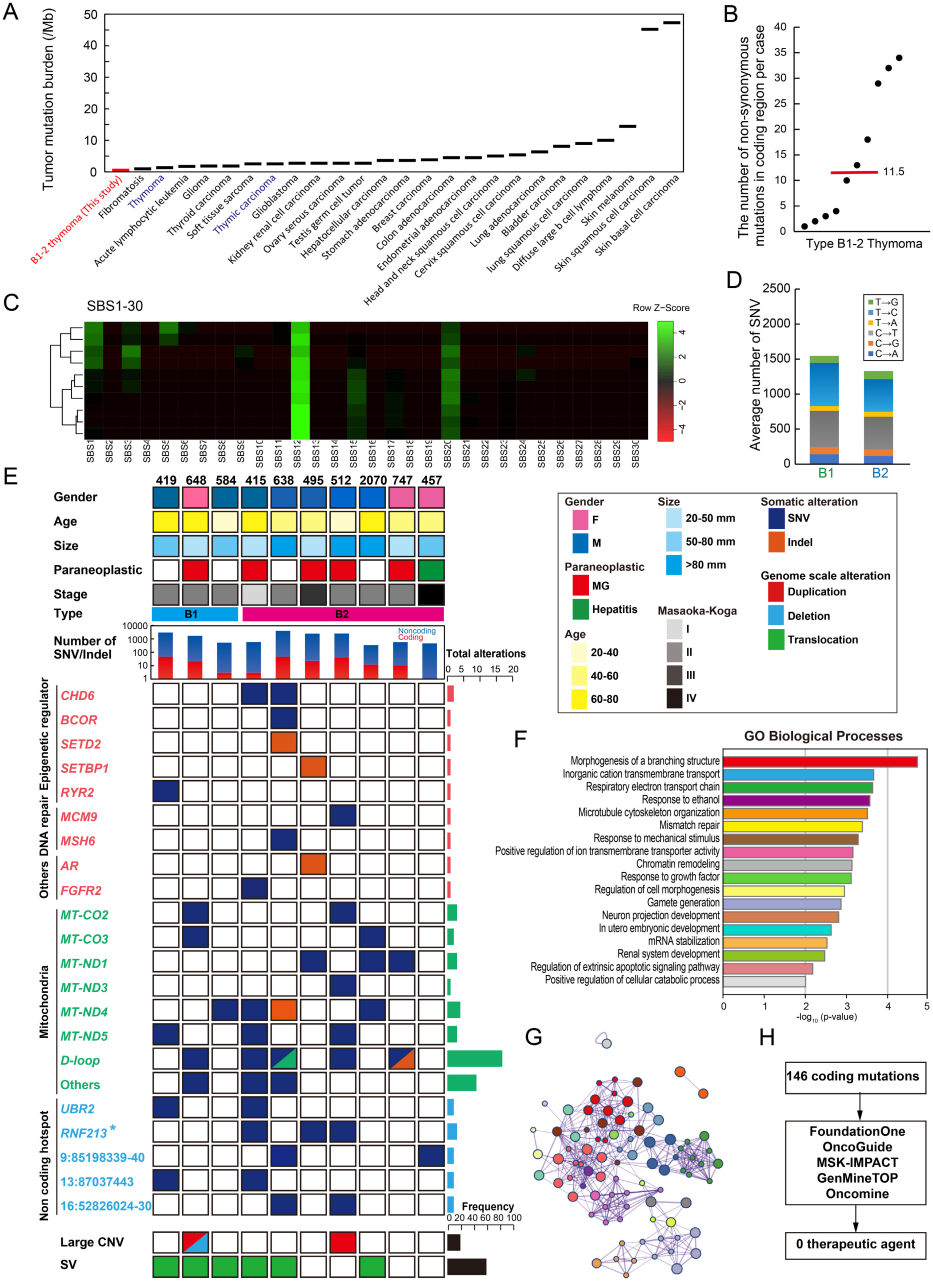
Identification of coding, noncoding, and mitochondrial mutations. (A) Frequencies of somatic mutations observed in protein‐coding regions. The *y*‐axis represents the median number of somatic mutations observed in each tumour, while the *x*‐axis indicates the tumour type. Tumour types are ordered by the number of somatic mutations. (B) Distribution of coding mutation burdens per patient in type B1 and B2 patients. The number of somatic DNA variants per patient is depicted along the *y*‐axis, with each dot representing an individual thymoma patient. The red‐coloured bar represents the median value. (C) Mutational signatures. Unsupervised clustered heatmap of contributions from 30 mutational signatures (SBS1–30) for thymomas. (D) Comparison of six base‐substitution patterns between B1 and B2 thymomas. (E) Detection of somatic mutations in type B1 and B2 thymomas. The main plot shows information for coding and noncoding regions with mutations in 10 thymomas. Mutations most likely to be related to thymoma pathogenesis are classified into seven categories, indicated on the left: I: epigenetic modifiers; II: DNA repair pathway; III: other genes; IV: mitochondria genes; V: noncoding hotspot; VI: large copy number variations; VII: structural variants. Colour key: blue, SNV; orange, Indel; red, duplication; light blue, deletion; green, translocation. The upper histogram displays clinical information (gender, paraneoplastic disease, age, tumour size, Masaoka–Koga classification, thymoma type) and total number of SNVs and indels. Asterisk (*): Case #512 has a coding mutation, while cases #415 and #495 have noncoding hotspot mutations. (F) Functional validation of frequently mutated genes in thymoma. The GO Biological processes were identified by enrichment analysis for thymoma‐associated genes. (G) Network analysis. The cancer‐related genes in our datasets were examined using Metascape to identify human‐curated pathway datasets. The size of the spheres indicates the quantity of genes detected in our cohort, and the colour of the spheres corresponds to the GO biological processes shown above. (H) Strategy to identify potential therapeutic agents based on the current companion diagnostics.

Deep sequencing was performed across entire genome regions. Sequencing depths for tumours and corresponding normal components were 115× and 129× on averages, respectively, which were greater than those in typical WGS studies (supplementary material, Table S2). Comparison of tumour cells with normal cells showed that all thymoma samples, including the pT3 samples, had a low TMB (mean 0.42 mutations/Mb in a protein‐coding region, range 0.03–0.97). The TMB of type B1‐2 thymoma was the lowest among the various tumours, with 40% of cases having four or fewer mutations observed in the coding region (Figure 2A,B).

The nucleotide substitution pattern of mutations assists in clarifying the background of tumorigenesis. Mutation signatures were successfully determined in all cases. The spectrum of base substitutions showed a strong predilection for T > C and C > T transitions, and it was similar to the known cancer mutational signature (single‐base substitution 12: SBS12) reported by global cancer studies (Figure 2C) [31]. This pattern is reported in a small percentage (< 20%) of hepatocellular carcinomas, but the exact aetiology is still unclear.

No specific differences were detected between type B1 and B2 thymomas with respect to the proportion of coding/noncoding SNVs, damaging/benign mutations, and mutation signatures (Figure 2D; supplementary material, Figure S2).

### Paucity of coding mutations in the nuclear genome

Exon analysis detected somatic mutations in epigenetic regulators (*CHD6*, *BCOR*, *SETD2*, *SETBP1*, *RYR2*), DNA mismatch repair genes (*MCM9*, *MSH6*), collagen‐related genes (*COL4A1*, *COL6A3*, *COL6A5*), and some additional genes, such as *AR* and *FGFR2* (Figure 2E and supplementary material, Table S3). GO analysis showed that the mutated genes were collectively involved in chromatin remodelling, DNA repair, stress response, organ development, and cell morphology (Figure 2F,G; supplementary material, Figure S3). Gene mutations previously reported in type A and AB thymoma (e.g., *GTF2I*, *HRAS*, *KRAS*) [9, 10, 32, 33, 34] were not identified in our cohort. *TP53*, *KIT*, and *CDKN2A* are frequently mutated in thymic carcinoma [9, 10], but were wildtype in the type B1 and B2 thymomas examined. Using the gene companion diagnostic approach currently used in clinical practice, we attempted to identify potential therapeutic targets based on detected protein mutations. However, after applying five different panel testing algorithms, no candidate therapeutic agents were identified as of May 2024 (Figure 2H). Synonymous mutations were detected less frequently than nonsynonymous mutations (1 versus 2.2) and were not recurrent (supplementary material, Table S4 and Figure S4).

### Comprehensive profiling of noncoding mutations

**Figure 3:**
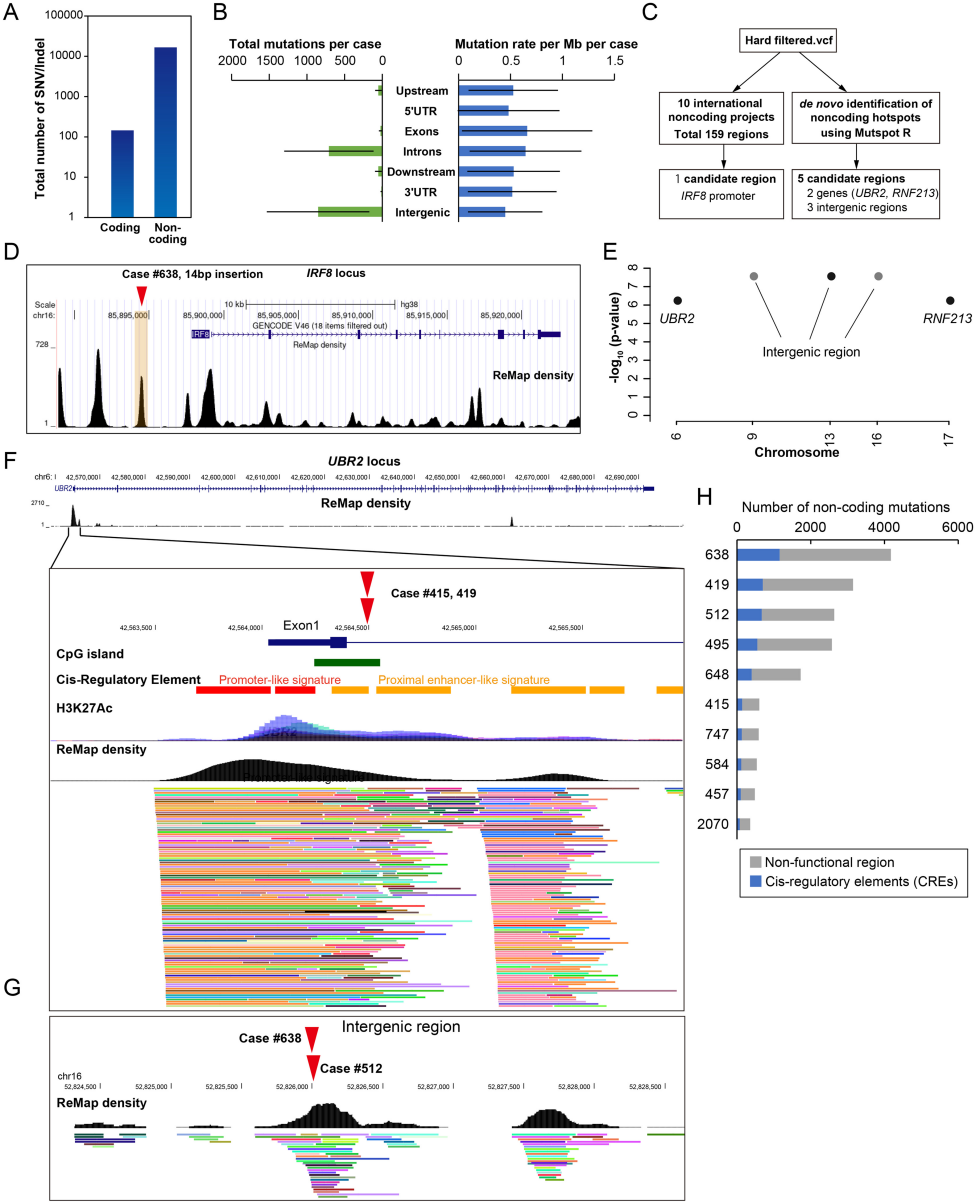
Identification of noncoding mutations. (A) Comparison of the total number of mutations in the coding versus noncoding regions. (B) Total number of mutation events (SNV + indel) and mutation rates (total events per Mb per patient) in different functional regions. (C) Strategy to identify significantly mutated noncoding regions. (D) Identification of mutations in the *IRF8* promoter based on a list of known hotspot regions, which are detected in ten international projects. (E) Manhattan plot of recurrent noncoding mutations. The plot was generated using MutSpot from whole‐genome sequencing data. Only hotspots with FDR < 0.05 are plotted. Mutations are located in the region characterised by Remap density (black). (F) Noncoding mutations in *UBR2* locus and its genomic locus characterisation. The *UBR2* mutation is located within the enhancer, and is characterised by the presence of CpG islands and H3K27ac. Genomic features are coloured as follows: gene structure (blue), CpG island (green), putative promoter (red), putative enhancer (orange), H3K27Ac (purple and blue), Remap density (black). (G) Mutations in the intergenic region and its genomic locus characterisation. Mutations are located in the region characterized by Remap density (black). (H) Number of mutations in cis‐regulatory elements. The noncoding mutations are divided into two regions: nonfunctional region (grey) and cis‐regulatory elements (CREs; blue).

A median of 11.5 exonic nonsynonymous alterations was observed per case. Notably, 40% of cases harboured four or fewer genetic mutations in the protein‐coding region (Figure 2B). These findings underscore the necessity to focus on noncoding regions. To investigate the noncoding landscape of type B1‐2 thymomas, we conducted a genome‐wide survey of noncoding regions with an average coverage of 39.4× (range: 30.3× −65.1×) (supplementary material, Table S2). The mean total number of somatic mutations in noncoding regions per case was 1,671.3 (range: 348–4,164), which was 79.2 times (range: 29.0–484.0) more frequent than in coding regions in type B1‐2 thymomas (Figure 3A). The majority of noncoding mutations were observed in introns (41.6%) or intergenic regions (44.6%), yet the mutation rate per Mb were comparable across the seven different genomic regions (range: 0.45–0.64/Mbp) (Figure 3B).

Two distinct methodologies were employed to identify the recurrent noncoding mutations. In the initial approach, we conducted a search for the previously identified recurrently mutated regions, retrieved from ten international projects (Figure 3C). A total of 159 regions have been listed as significantly mutated regions, and the *IRF8* promoter region was mutated in our study (Figure 3D; supplementary material, Table S4).

In a second approach, we applied *de novo* identification of noncoding hotspots using the MutSpot R‐package on the entire WGS dataset (Figure 3C). This computational workflow identified five recurrent mutations: two E3 ubiquitin‐protein ligase (*UBR2* and *RNF213*) loci and three intergenic regions (Figure 3E; supplementary material, Figure S5 and Table S4). The mutation in the *UBR2* locus was identified in the CpG‐rich enhancer in Intron 1 (Figure 3F). Mutations in the *RNF213* locus were identified in the last intron (Intron 67) in two cases and in the coding mutation (Exon 67) in another case (supplementary material, Figure S5). The intergenic region on chr16 52826024–52826030 displayed regulatory signals, suggesting the potential significance of this region at the transcriptional regulatory level (Figure 3G). A systematic noncoding analysis identified 405.0 mutations per case on putative cis‐regulatory elements, while 1,266.3 mutations were classified as nonfunctional (Figure 3H).

### Copy number and structural variants

**Figure 4:**
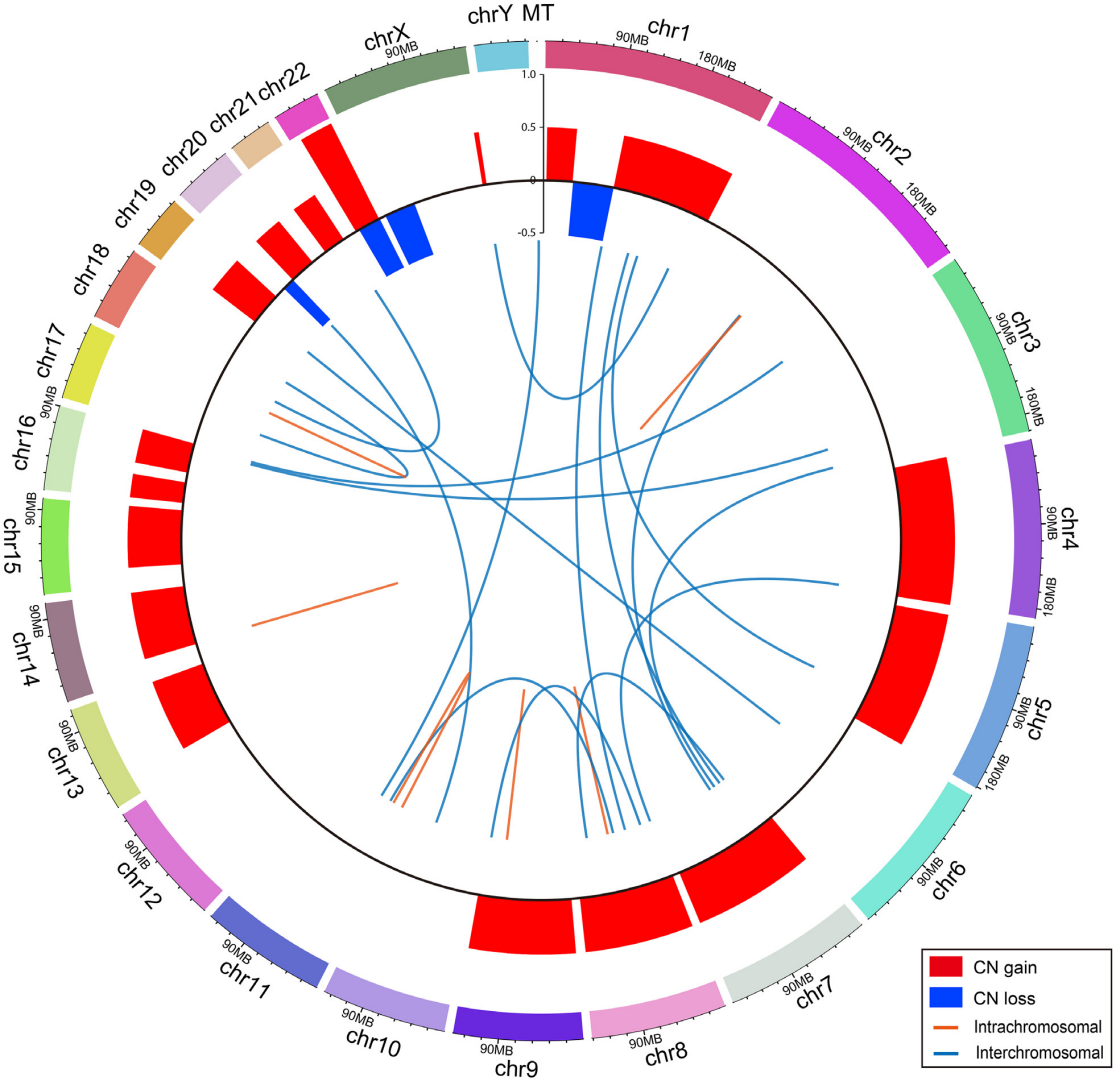
Genomics landscape of copy number and structural variants in type B1 and B2 thymoma. Circos plot for large‐scale genomic alterations in 10 thymomas. Copy number events are summarized in the outer circle, with red indicating copy number gains and blue indicating copy number losses. Translocations are marked with blue lines for interchromosomal events and orange lines for intrachromosomal events. For intrachromosomal translocations, the orange connecting line may appear as a single straight line if the joined regions lie within 1 Mbp.

Arm‐level somatic copy number alterations were detected in 20% of cases (Figure 4; supplementary material, Figure S6 and Table S5). An average of 2.3 fusion events were identified per patient, with 60% of patients having translocations, including 1.7 interchromosomal fusions and 0.6 intrachromosomal fusions (Figure 4; supplementary material, Figure S6 and Table S6). None of these translocations have been previously reported. The *MAML2* gene fusions (*KMT2A*::*MAML2*, *YAP1*::*MAML2*, and *CRTC1*::*MAML2*), which have been observed in a small subset of thymic epithelial tumours, were not identified in our cohort [11, 35, 36, 37].

### Tumour‐specific/enriched mitochondrial heteroplasmic shift

**Figure 5:**
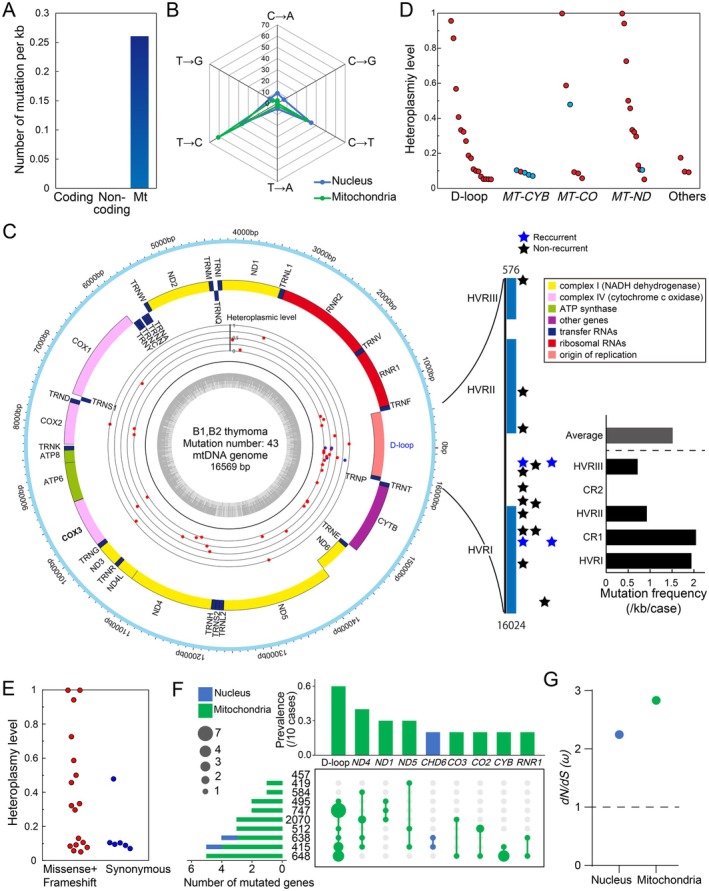
Somatic mtDNA mutations in thymoma patients. (A) Comparison of somatic mutation rates in the coding, noncoding, and mitochondrial regions. (B) Comparison of seven base‐substitution patterns between nuclear and mitochondrial genomes. (C) Circos plots showing the distribution of mtDNA somatic mutations in thymoma. Somatic mutation events are summarized in the inner circle, with blue indicating recurrent mutations. The right vertical bar shows the distribution of D‐loop mutations. HVR: Hypervariable region, CR: conserved region. (D) Heteroplasmy levels of each mtDNA mutation in five different functional regions. (E) Comparison of heteroplasmy levels of each mtDNA mutation between nonsynonymous and synonymous mutations. (F) Summary of genes commonly mutated in multiple cases. Upset plots showing the prevalence of each gene mutation. Blue indicates mutations in the nuclear genome, while green indicates mutations in the mtDNA. (G) Comparison of *dN/dS* (*ω*) between nuclear and mitochondrial coding mutations.

In thymoma, genomic alterations in mitochondrial DNA (mtDNA) have not previously been investigated. We conducted a survey of mtDNA sequences with an average coverage of 368× (range: 71× −1,244×) (supplementary material, Table S2). An average of 4.3 mtDNA mutations per patient were detected predominantly in tumour fraction, with 90% of patients having mutations (Figure 5; supplementary material, Table S7). The mutation density (number of mutations per length of kb) in mtDNA was ~500 times higher than that in nuclear genome (0.26/kbp versus 0.0005/kbp) (Figure 5A). The mutation signature revealed a strong bias towards T > C transitions in mitochondria, as well as in the nucleus (i.e. SBS12 pattern), suggesting that similar mechanisms may contribute to mutation accumulation between nuclear and mitochondrial genomes (Figure 5B).

The D‐loop region was the most frequently mutated (*n* = 17), followed by *MT‐CYB* (*n* = 5), *ND4* (*n* = 5), *ND1* (*n* = 3), *ND5* (*n* = 3), *CO2* (*n* = 3), and other regions (*n* = 7) (Figure 5C; supplementary material, Table S7). Hypervariable region I (HVR I) and its surrounding regions showed a high density of genetic mutations, with two recurrent mutations detected (m.16258 A > G and m.16527 C > T). The transmission of mitochondrial DNA to the nuclear genome (Chr 11) was also detected in tumour cells (Figure 4). Heteroplasmy levels were similar between different genes, but different between nonsynonymous and synonymous mutations (Figure 5D,E). A summary of genes commonly mutated in multiple cases revealed that only the *CHD6* gene was mutated in the nuclear genome, while eight genes were mutated in the mitochondrial genome (Figure 5F). The *dN/dS* ratio (*ω*) of protein‐coding genes was greater than 1 in both the mitochondria and nuclei, and was higher in mitochondria than in nuclei (Figure 5G).

**Figure 6:**
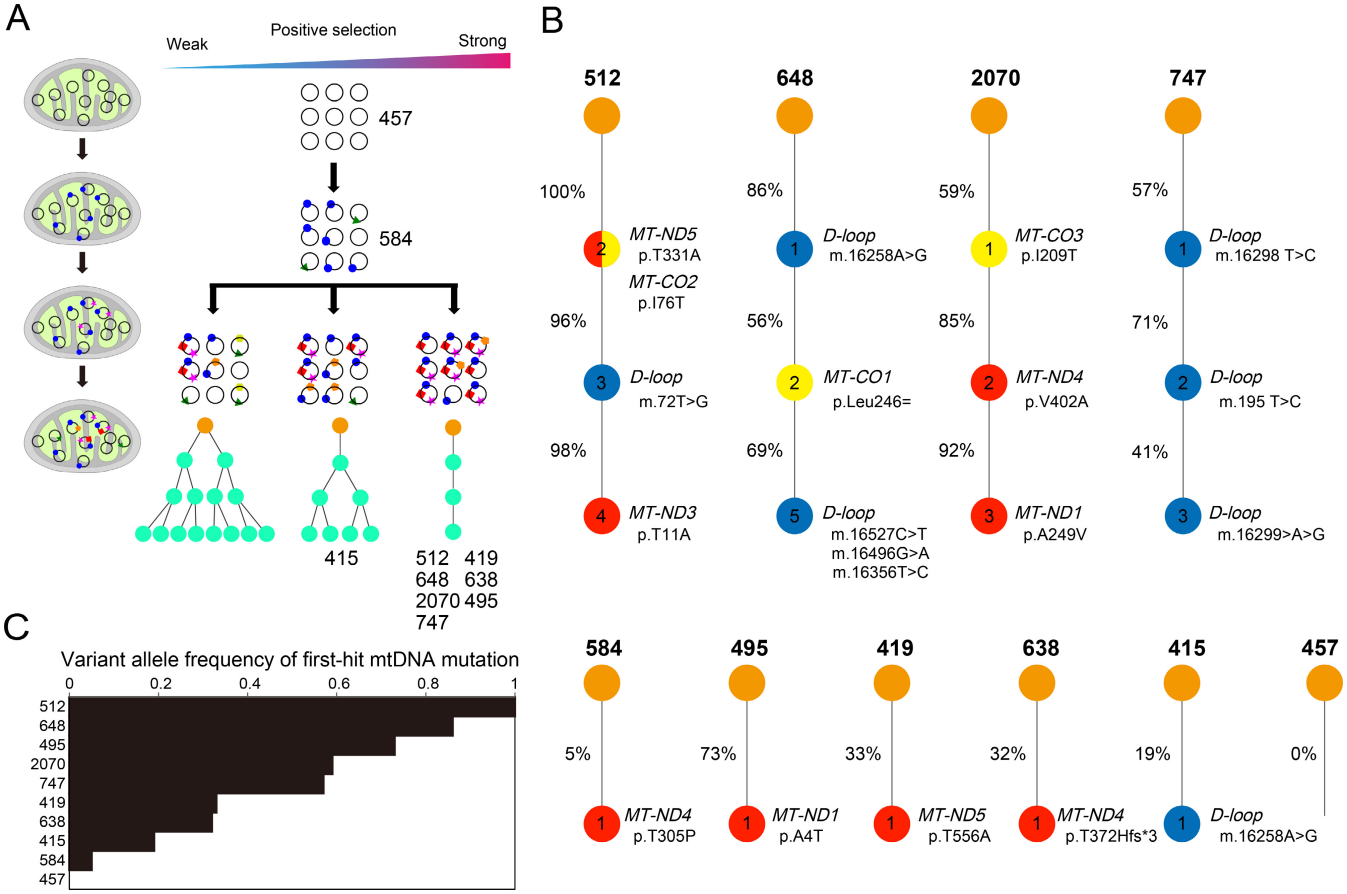
Evolutionary lineages of mitochondrial DNA mutations. (A) Evolutionary scenarios of mitochondrial mutations. Exposure of normal cells to carcinogenic agents results in the acquisition of random mutations in mtDNA. The mutations frequency can increase or decrease in tumour cells, depending on the biological effect. Ten cases are assigned to this panel. This figure was created using BioRender (https://BioRender.com). (B) Clonal evolution with mtDNA mutations at each step shown. Numbers inside the nodes represent the total number of mutations for the corresponding clone, and percentages next to the nodes (clones) show the proportion of each clone. Red, *MT‐ND* genes; Blue, *D‐loop*; yellow, *MT‐CO* genes. (C) Variant allele frequency of the first‐hit mtDNA mutation.

When we traced the evolutionary lineage of mtDNA mutation, most cases could be explained by a linear evolutionary model, suggesting that positive selection may have been operating on the mitochondria genome during thymoma development (Figure 6A,B). The VAF of putative first‐hit mutations were often 50% or more (Figure 6C). D‐loop mutations were the most frequent, with 3 out of 10 primary mutations, followed by some *MT‐ND* and *MT‐CO* genes (supplementary material, Figure S7).

## Discussion

Comprehensive identification of somatic mutations that contribute to tumourigenesis is a priority of tumour sequencing projects. To date, the majority of studies have focused on mutations within protein‐coding regions of the genome [38]. Nevertheless, several recent discoveries, including the identification of recurrent somatic mutations in the *TERT* promoter in various cancer types, indicate that mutations in noncoding regions are also crucial in tumour development [39]. Moreover, the analysis of whole‐genome sequencing data from tumours has elucidated novel mutational patterns and processes imprinted on cancer genomes [31].

Thymomas have been reported to have very few coding mutations [12]; therefore, it is essential to prioritise the analysis of noncoding regions in genomic research. In particular, type B1 and B2 thymomas are predicted to have low numbers of coding mutations [10]. The genetic landscape of type B1 and B2 thymomas is less characterized compared to other cancers and other types of thymomas because genetic investigation is hampered by the abundance of nontumorous immature T lymphocytes. Also, deep WGS analysis is five times more expensive than whole‐exome analysis [40].

In this study we developed new methods that utilize an automated image cell sorter in combination with CD45 and CD205. Our approach achieved 100% purity of tumour cells and complete elimination of immature lymphocytes. This methodology enabled molecular profiling of pure tumour cells from Type B thymoma tissues and can serve as a basis for thymoma research. The current limitations of this approach include the fact that the DEPArray system is not readily available at all institutions and that the experimental setup is not straightforward, time‐consuming, and labour‐intensive.

We have newly identified five noncoding regions that are repeatedly mutated in type B1‐2 thymomas. One of the identified regions is the *RNF213* locus, a large E3 ubiquitin ligase with a dynein‐like core involved in a distinct ubiquitin transfer mechanism. The *RNF213* gene is mutated in some malignant tumours, including gastric cancer, ovarian cancer, lung cancer, liver and bile duct cancer [41, 42, 43], yet there have been few functional studies on *RNF213* mutations in malignancies. Previous reports have indicated that *RNF213* acts as a tumour suppressor and its mutation promotes tumour development and progression [41, 42, 43, 44]. To determine whether the putative causal mechanism is disease‐specific, it will be necessary to compare these mutations to the thousands of noncoding region mutations in other cancers that have been analysed in international projects. However, these have not been compiled into a database or published as supplemental data, and a database that is easily accessible to all researchers is needed.

Another mutated region is the *UBR2* locus, a RING E3 ligase that regulates gene expression and chromatin‐associated ubiquitylation in response to DNA damage. *UBR2* is widely expressed in a variety of human cancer tissues, particularly breast cancer, prostate cancer, and lymphomas [45]. This gene has been demonstrated to promote tumour growth and metastasis [45]. In addition to proteasomal degradation, *UBR2* has been shown to regulate the Erk/MAPK pathway, thereby preventing caspase‐independent cell death [46]. In this study, two E3 ubiquitin ligases were identified. Malignant tumour cells exhibit a high dependence on the proteasomes, and it may be possible to exploit this dependence to induce cell death using proteasome inhibitors.

It is unclear from our study whether the identified noncoding mutations are functional. It is possible that mutations in noncoding regions are less functional than those in coding regions, and in contrast, some mutations in cis‐regulatory regions may regulate the expression of a large number of genes and have a significant impact on cellular homeostasis. The Remap density illustrated in Figure 3 serves as an indicator of the function of noncoding regions and suggests that a large number of transcription factors bind to a single cis‐regulatory region. Nevertheless, these data vary widely among diverse cell types and among research methods. Furthermore, given the interindividual differences among patients and the strikingly diverse patterns of mutations, functional analysis using thymic epithelial cells or thymoma cells would be essential to confirm the function of each noncoding mutation. The function is also unknown for fusion genes and chromosomal translocations. Further studies using RNA‐seq are needed to confirm the structure of the fusion gene. If the codon reading frame is shifted, it will mean a loss of function, and if a fusion gene is formed, it is expected to acquire a new function.

In this research, we did not focus on synonymous coding mutations because we were unable to provide any evidence that these mutations are significantly involved in thymoma development. Additionally, we did not identify the recurrent synonymous mutation. It is known from previous reports that these mutations can affect mRNA secondary structure and stability, the rate of translation, and post‐translational modifications of nascent polypeptides [47, 48, 49]. In our study, an average of 6.5 gene mutations were identified per case, although the number was smaller than that of nonsynonymous substitutions. This question should be addressed in the future through the development of comprehensive analyses of synonymous mutations, in combination with computational methods for mRNA secondary structures. In addition, epigenetic abnormalities represent the next target for analysis of thymoma, and should be investigated using a variety of methods, such as immunostaining, methylation beadchip, assay and whole‐genome bisulfite sequencing [50].

It is widely recognised that mtDNA mutations play an important role in the initiation and progression of malignant tumours [51]. However, there are no reports of a mitochondrial heteroplasmic shift in thymomas and thymic carcinomas. This study provides the first systematic and comprehensive profiling of mtDNA mutations in thymoma cells, with the following advantages: (i) The average NGS coverage depth of more than 360‐fold enabled accurate detection of mtDNA mutations with heteroplasmy levels as low as 5%; (ii) the data from matched control and thymoma tissues allowed accurate identification of thymoma‐associated mtDNA mutations; (iii) DEPArray‐based tumour cell isolation ensured the validity of mtDNA mutation profiling in thymoma tissues.

Despite the small size of the mitochondrial genome, 43 mutations have been detected in ten thymomas, and the mutation number is comparable to that in other malignancies (4.3 versus 3 per patient) [52]. The mutation rate in the mitochondrial genome was also reported to be 55 times higher than that of the autosomes in prostate [53]. The reason for this higher mutation rate than the nuclear genome is presumably the lack of protective histones, the generation of reactive oxygen species in the inner membrane, and limited repair mechanisms that make mtDNA particularly vulnerable to damage [54]. A significant proportion of these mutations were located in the D‐loop, with heteroplasmy levels ranging from 5% to 96%. Consequently, our findings strongly underscore the pivotal role of the D‐loop region mutations in the pathogenesis of thymic epithelial tumours. The significance of the D‐loop has also been reported for several other malignancies, including breast cancer and hepatocellular carcinoma [55, 56]. Previous reports have also indicated that D‐loop mutations with high levels of intratumoural recurrence in cancerous tissues also have high levels of heteroplasmy. These findings indicate the possibility of positive selection for D‐loop and other mtDNA mutations in tumourigenesis. This is consistent with previous findings that cancer mutations predicted to have a large functional effect are significantly enriched and can be distinguished from passenger mutations with low functional effect [57]. This is also consistent with our findings that heteroplasmy levels are lower for synonymous than nonsynonymous mutations. In conclusion, these results provide compelling evidence supporting the crucial role of mtDNA mutations, particularly those in the D‐loop region, in thymoma tumourigenesis.

It has been reported that D‐loop alterations are significantly associated with reduced mtDNA content in malignant tumours and poor patient prognosis. Mouse with mitochondrial mutator phenotype, generated by a proofreading‐deficient version of DNA polymerase gamma (*Polg*), exhibited an accumulation of mtDNA mutations, including D‐loop mutations, and a reduction in mtDNA copy number [58]. In addition, mutant *Polg* was shown to markedly enhance the invasive potential of breast cancer cells *in vitro* [59]. Together these results, and the findings presented in Figure 5 indicate that D‐loop mutations may contribute to the malignant transformation of thymic epithelial cells by reducing mtDNA copy number. This hypothesis should be confirmed in future studies by developing mtDNA editing technology that can generate site‐specific mtDNA mutations [60], particularly in the D‐loop region.

The mutation signature was also investigated in this study. SBS12 was detected in all cases of type B1 and B2 thymomas. SBS12 has previously been reported in hepatocellular carcinomas and renal carcinomas [31, 61]. A recent report indicated that the proportion of SBS12 may vary geographically [61]. These findings indicate that exposure to agents contributing to SBS12 mutations in liver and kidney cancer is prevalent in certain countries. The specific agent responsible for SBS12 remains unknown and warrants further investigation. Nevertheless, the precedent set provided by other mutation signatures involving transcriptional strand bias suggests that the agent is probably exogenous.

Like all cancer genomics studies, our study has limitations. First, we analysed a relatively small number of type B1 and B2 thymomas. Further case accumulation and mutation validation are needed. Still, this represents the first cohort to date of thymomas analysed by deep whole‐genome sequencing followed by noncoding mutation analysis. Moreover, we developed a method to analyse the thymoma cells at highest tumour cellularity by combining DEPArray and CD205. The design of such whole‐genome sequencing studies was determined by balancing the number of samples with the complexity of noncoding genomic analysis. In this study, we chose to comprehensively analyse noncoding sequences, but the sample size was not large. Second, mutation calling was performed using the latest version of the genome analysis platform, although there are certain limitations in the accuracy of mutation detection. In particular, mutations in the centromere, repeat sequences, and pseudogene remain difficult to identify. An additional concern is contamination of nuclear‐mitochondrial segments (NUMTs), which has been the focus of attention in the last decade. Although we attempted to reduce the contribution of variation from mitochondrial DNA sequences present in NUMTs, it is impossible to completely eliminate contamination as long as the sequences are the same. To be sure, we estimated the extent to which NUMT affects mutation determination by referring to previous papers [62] and finally concluded that it does not significantly affect the interpretation (less than 3% maximum) from the theoretical calculations (see Supplementary materials and methods, section ‘Estimation of NUMT contamination in mitochondrial genome alignment’). Third, we intended to cover the entire genome, but there are regions with low read depth in either normal or tumour cells. Therefore, not all genetic mutations can be detected. To identify such mutations, it is necessary to perform ultra‐deep NGS and/or add long read NGS.

In conclusion, numerous noncoding and mtDNA mutations were identified in type B1 and B2 thymomas. Given the paucity of coding mutations detected in this disease entity, it is possible that other factors such as noncoding/mitochondria DNA mutations and epigenetic dysregulation may contribute to the development of thymoma. Targeting dysregulated signalling pathways and cellular processes involved in thymoma progression, such as ubiquitination and mitochondrial heteroplasmic shift, may be a promising approach for precision medicine in thymoma therapy. Furthermore, the combination of CD205 and CD45 with the DEPArray system proved to be useful for genetic analysis of thymoma. Continued efforts to unravel the genetics of thymoma through our approach will advance our understanding of thymoma biology and improve patient care.

## Author contributions statement

KF and DN conceived and designed the work. KF, IC, SH, KW, JK, EM, SN, AB and DN performed the experiments and obtained the data. KF, IC and DN analysed and interpreted the results. KF and DN drafted the article. KF, YT, MK and DN edited and revised the article. All authors approved the final version of the article.

## Supporting information


**Supplementary materials** **and methods**
**Figure S1**. Expression of CD45 and CD205 in thymoma tissue
**Figure S2**. Comparison of somatic alterations between type B1 and B2 thymomas
**Figure S3**. GO analysis
**Figure S4**. Comparison of synonymous and nonsynonymous mutations
**Figure S5**. Recurrent noncoding mutations
**Figure S6**. Copy number and structural variants in Type B1 and B2 thymoma
**Figure S7**. Frequency of primary and secondary hit mutations in the mitochondrial genome

**Table S1.** Background of patients (provided as separate Excel file)
**Table S2**. Evaluation of deep whole‐genome sequencing quality metrics (provided as separate Excel file)
**Table S3**. List of coding nonsynonymous mutations (provided as separate Excel file)
**Table S4**. List of coding synonymous mutations (provided as separate Excel file)
**Table S5**. List of recurrent noncoding mutations (provided as separate Excel file)
**Table S6**. List of large copy number alterations (provided as separate Excel file)
**Table S7**. List of chromosomal translocations (provided as separate Excel file)
**Table S8**. List of mitochondria DNA mutations (provided as separate Excel file)

## Data Availability

Data are available from the corresponding author upon reasonable request.
