# Supplementary material for: Genome‐wide analysis of somatic noncoding mutation patterns and mitochondrial heteroplasmic shift in type B1 and B2 thymomas
Source: J Pathol. 2025 Dec 4;268(2):135–49. doi: 10.1002/path.6496 (PMC12805629; doi:10.1002/path.6496)
Supplement: Supplementary file 1 — Supplementary materials and methods Figure S1. Expression of CD45 and CD205 in thymoma tissue Figure S2. Comparison of somatic alterations between type B1 and B2 thymomas Figure S3. GO analysis Figure S4. Comparison of synonymous and nonsynonymous mutations Figure S5. Recurrent noncoding mutations Figure S6. Copy number and structural variants in Type B1 and B2 thymoma Figure S7. Frequency of primary and secondary hit mutations in the mitochondrial genome [file PATH-268-135-s002.docx]

**Genome-wide analysis of somatic noncoding mutation patterns and mitochondrial heteroplasmic shift in type B1 and B2 thymomas**

K Fujikura *et al. J Pathol* <https://doi.org/10.1002/path.6496>

**Supplementary materials and methods**

**Supplementary Figures S1–S7**

**Supplementary Tables S1–S8**

Reference numbers refer to the main text list

**Supplementary materials and methods**

**Estimation of NUMT contamination in mitochondrial genome alignment**

Nuclear-mitochondrial segments (NUMTs) are transpositions of mitochondrial DNA into the nuclear genome in eukaryote. NUMT sequences are almost identical to the mitochondrial genome, except for the junction sequence, and thus may have a significant impact on the mitochondrial genome alignments and somatic mutation detection. Previous reports indicate that more than 99% of individuals had at least one NUMT [62]. However, each NUMT fragment is relatively short and mitochondria copy number is high compared to the nuclear genome (> 200 copies versus 2 copies), and therefore NUMTs may not pose a major problem in detecting somatic mutation in the mitochondrial genome. In this study we performed a calculation based on data from the literature to determine how much NUMTs affect mitochondrial alignment. From the latest paper, it is estimated that the median length of NUMTs an individual has is 156 bp and each person has 4.7 NUMTs [62]. From these data, two formulas are derived:

1. Total length of NUMTs per individual = 156 bp × 4.7 = 733 bp
2. Percentage of NUMTs in mitochondrial genome alignment (i.e. contamination level) = 733 bp (NUMT) / 16,549 bp (MT genome) / difference in read depth (≒ copy number) × 100

< 0.001–3%

Since mitochondria has 16,549 bp of DNA and a higher copy number than the nuclear genome, we consider this a level of contamination that does not significantly affect the overall interpretation, although the above calculations are theoretical.

**Frozen tissue processing**

Frozen sections (60 µm thickness) were fixed in 1 ml of 2% paraformaldehyde for 15 min at room temperature. The sections were then washed four times with 1 ml of PBS and centrifuged at 800×g for 5 min. The samples were digested in a solution of 5 ml of a mixture of collagenase (Sigma-Aldrich, St. Louis, MO, USA) and dispase (Gibco, Grand Island, NY, USA), with each enzyme present at a concentration of 0.1% in RPMI 1640 (Gibco), for a period of 15–30 min at 37 °C. Subsequently, 5 ml of RPMI 1640 with 10% foetal calf serum (Gibco) were added together with EDTA to a final concentration of 2 mM. Then the samples were centrifuged at 460×g for 10 min, after which the pellets were resuspended in 2 ml of FACS buffer (1% BSA and 2 mM EDTA in PBS) and filtered through a 35-µm mesh. The samples were centrifuged at 460×g for 10 min and resuspended in 1 ml of FACS buffer. The cell number was then counted, and a total of 1–2×10^5^ cells were stained in 100–200 μl of FACS buffer. After incubation with Fc block (BioLegend, San Diego, CA, USA) for 10 min at room temperature, APC anti-human CD45 (clone HI30; BioLegend) and PE anti-human CD205 (clone HD30; BioLegend) antibodies were added and incubated for 20 min at 4 °C. After 20 min of incubation, 2 μl of a 0.1 mg/ml stock of Hoechst (Thermo Fisher Scientific, Waltham, MA, USA) were added to label the nuclei and samples were incubated for another 10 min at room temperature. Subsequently, the samples were washed with 2 ml of FACS buffer and pelleted at 460×g for 10 min. The cell pellets were resuspended in 200 μl of FACS buffer and stored at 4 °C.

**Cell isolation**

The isolation of normal and tumour cells was conducted using a DEPArray V.II system with A300K cartridges (Menarini Silicon Biosystems, Philadelphia, PA, USA). A total of < 1×10^5^ stained cells in FACS buffer were washed with 1 ml of DEPArray Buffer (Menarini Silicon Biosystems) and spun at 460×g for 10 min. The cell pellet was washed once more with 1 ml of degassed buffers and was resuspended in 30 μl of the same buffer. The number of cells was counted and the volume was adjusted to yield ~10,000 cells in 14 μl. The samples were then loaded into the cartridges following the manufacturer’s protocol. The selected cells were eluted as single cells using 200 μl PCR tubes for collection. The PCR tubes were centrifuged at 14,100×g for 30 sec, after which 100 µl of PBS were added to the tube and centrifuged at 14,100 ×g for 10 min. Subsequently, the supernatant was removed, leaving 1 μl of PBS with the pellet and stored at −20 °C until DNA amplification

**Genomic DNA amplification**

Genomic DNA was amplified using the Ampli1 WGA kit (Menarini Silicon Biosystems) following the manufacturer’s protocol. The quality of the amplified DNA was evaluated using the Ampli1 WGA QC kit (Menarini Silicon Biosystems). Only samples exhibiting four distinct bands were used for the genome sequencing. To obtain sufficient DNA for WGS, reamplification of the genomic DNA was needed. Reamplification and double-strand conversion was done using the Ampli1 ReAmp/ds Kit (Menarini Silicon Biosystems) following the manufacturer’s protocol. Reamplified DNA was purified before and after MseI-digestion of the amplification adaptors. Removal of WGA adaptors is recommended before sequencing library preparation in order to avoid unwanted unspecific product capture and sequencing. Purification was done with QIAquick PCR Purification Kit (Qiagen, Hilden, Germany) following manufacturer recommendations. Ampli WGA products are expected in the range of 0.1 to 2 kb. All the purified samples were analysed on TapeStation (Agilent Technologies, Santa Clara, CA, USA) using D5000Screen tapes. Some samples were also analysed using the High sensitivity DNA assay on the Bioanalyser (Agilent Technologies) to confirm the distribution of DNA fragments following the manufacturer’s protocol. The final quantification of the purified samples was conducted using the Qubit fluorometer reagents (Thermo Fisher Scientific) in the Glomax Discover plate reader (Promega, Madison, WI, USA).

**Deep whole-genome sequencing**

The whole-genome sequencing (WGS) library was constructed using the NEBNext Ultra™ II DNA Library (New England Biolabs, Ipswich, MA, USA). The sequencing libraries were then multiplexed and clustered on the flowcell. Subsequently, the flowcell was loaded onto the NovaSeq 6000 instrument (Illumina, San Diego, CA, USA). The samples were subjected to sequencing using a 2 × 150 paired-end configuration. Image analysis and base calling were conducted using the NovaSeq Control Software on the NovaSeq instrument (Illumina). The raw sequencing data (.bcl files) generated from the Illumina NovaSeq were converted into fastq files and demultiplexed using Illumina's bcl2fastq software. A total of at least 288 gigabases of raw read data was generated for all samples.

**Detection of copy number alterations**

The CNV workflow provided with the DRAGEN Somatic Platform (Illumina) was performed based on 150 bp fragments. The reads were counted in 500 kb bins, followed by GC bias correction and normalization against a reference set. Segmentation was conducted via Shifting Level Models with the disabled merging of two adjacent segments. The interactive Circos plot was generated using the R-package shinyCircos [63].

**Mutation signature**

Single base substitution (SBS) mutational signatures were extracted from the sample’s WGS data using deconstructSigs [64], with default parameter settings. The COSMIC V2 Mutational Signature Set [65] was used as the reference signature set.

**Gene companion diagnosis**

A search was conducted for gene mutations in the coding regions identified in this study against the list of genes and corresponding potential therapeutic agents listed in the five following gene companion diagnostic approaches: FoundationOne; OncoGuide; MSK-IMPACT; GenMineTOP; Oncomine.

**Immunohistochemistry**

Deparaffinized sections were heat-treated and incubated with the primary CD205 antibody (clone: 11A10, dilution 1:25; Novocastra, Newcastle, UK) as previously described [30]. Immunostaining for CD205 was performed on a Bond Max autostainer (Leica Microsystems, Wetzlar, Germany) following the manufacturer’s protocol.


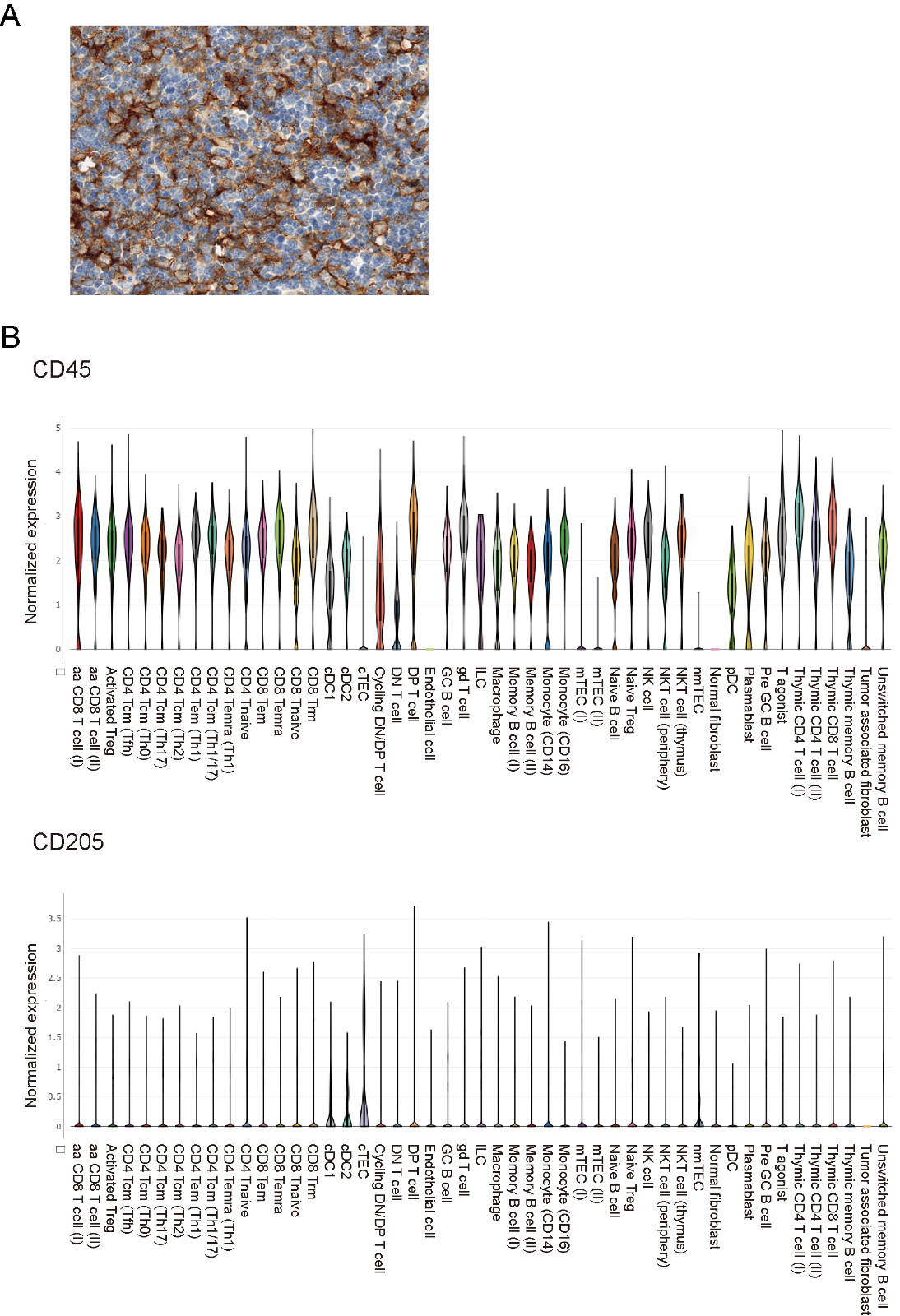


**Figure S1. Expression of CD45 and CD205 in thymoma tissue**. (A) Immunohistochemistry of CD205 in type B1 thymoma. (B) Droplet-based single-cell RNA sequencing (scRNA-seq) analysis of type B1 and B2 thymoma cells. Violin plots depicting expression levels of CD45 and CD205.


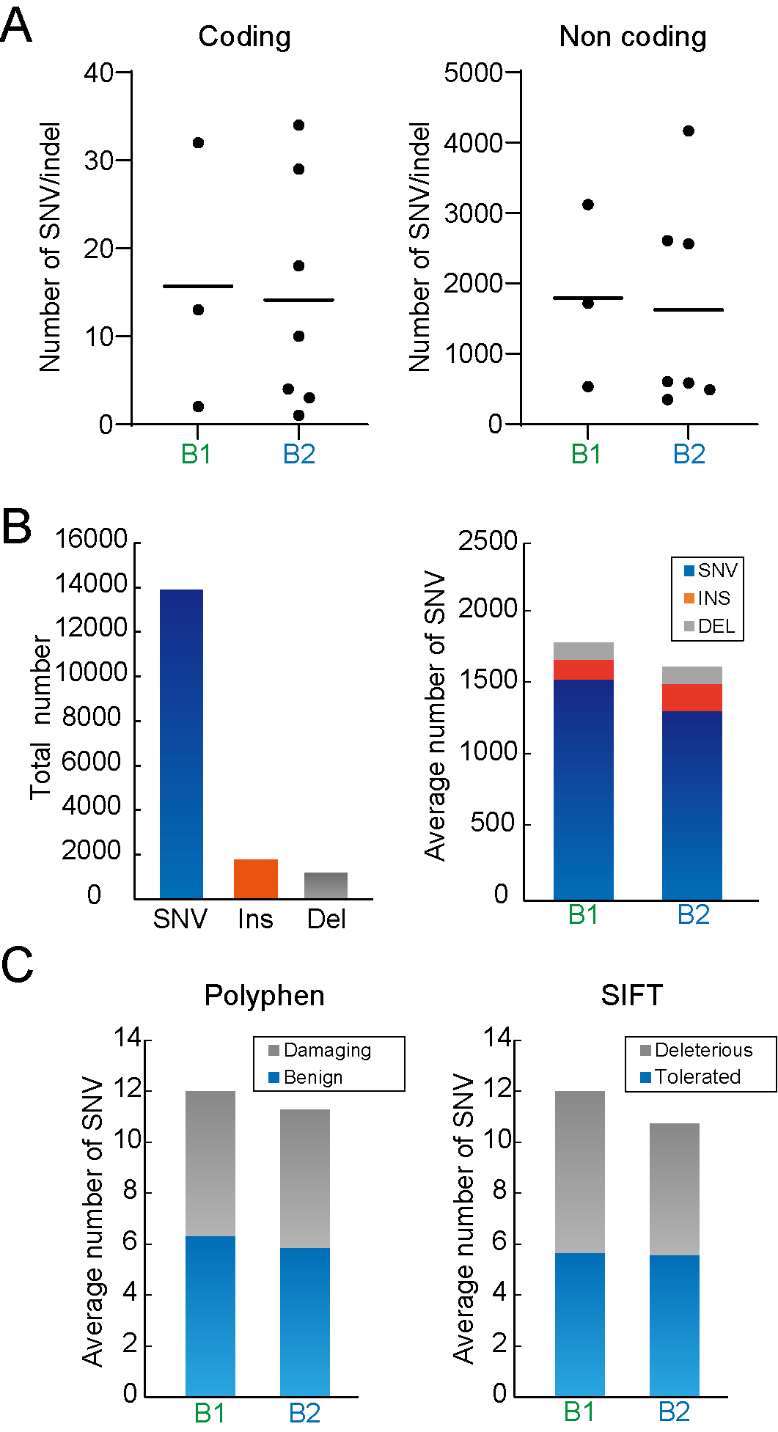


**Figure S2. Comparison of somatic alterations between type B1 and B2 thymomas.** (A) Coding and noncoding regions. (B) SNV, insertion, and deletion. (C) Polyphen and SIFT analysis.


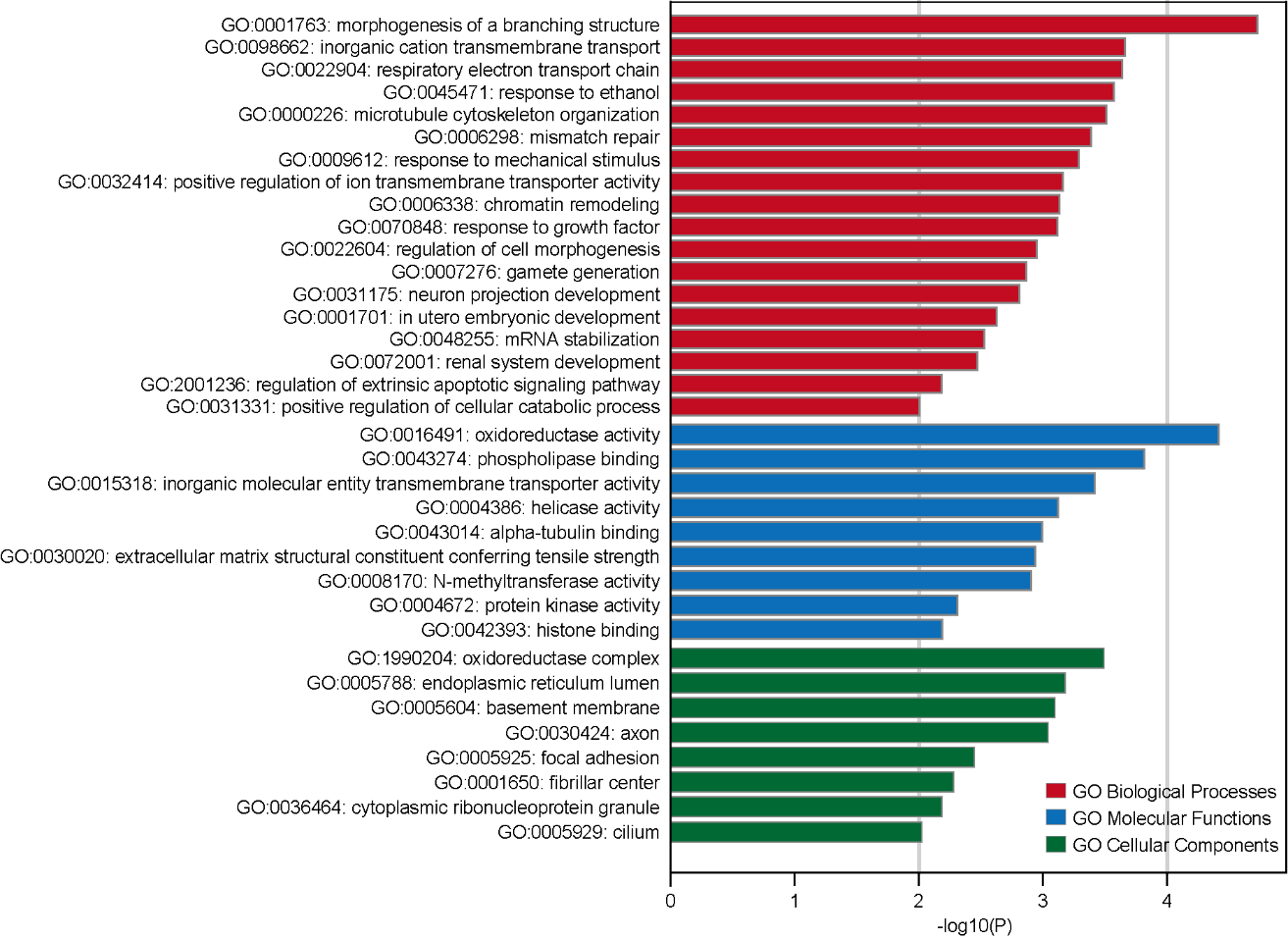


**Figure S3. GO analysis.** GO analysis for biological process, cellular component, and molecular function terms was performed on mutated genes; all terms with *p*< 0.01 are shown.


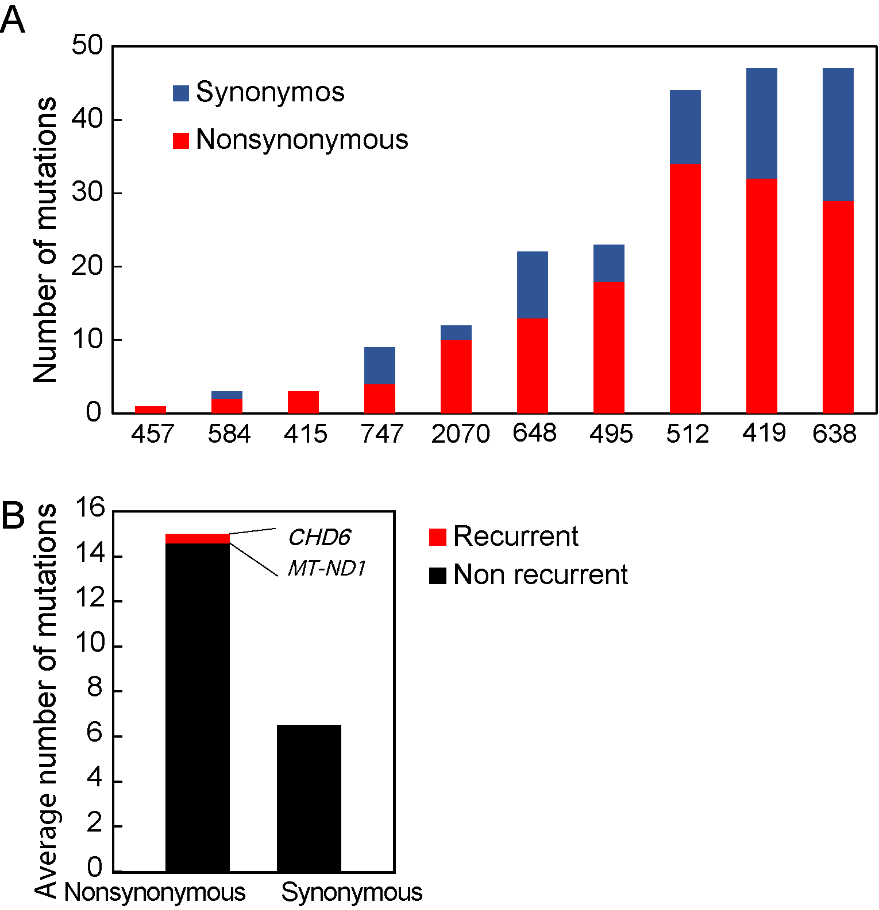


**Figure S4. Comparison of synonymous and nonsynonymous mutations**. (A) Number of synonymous and nonsynonymous mutations in each case. (B) Recurrent mutations.


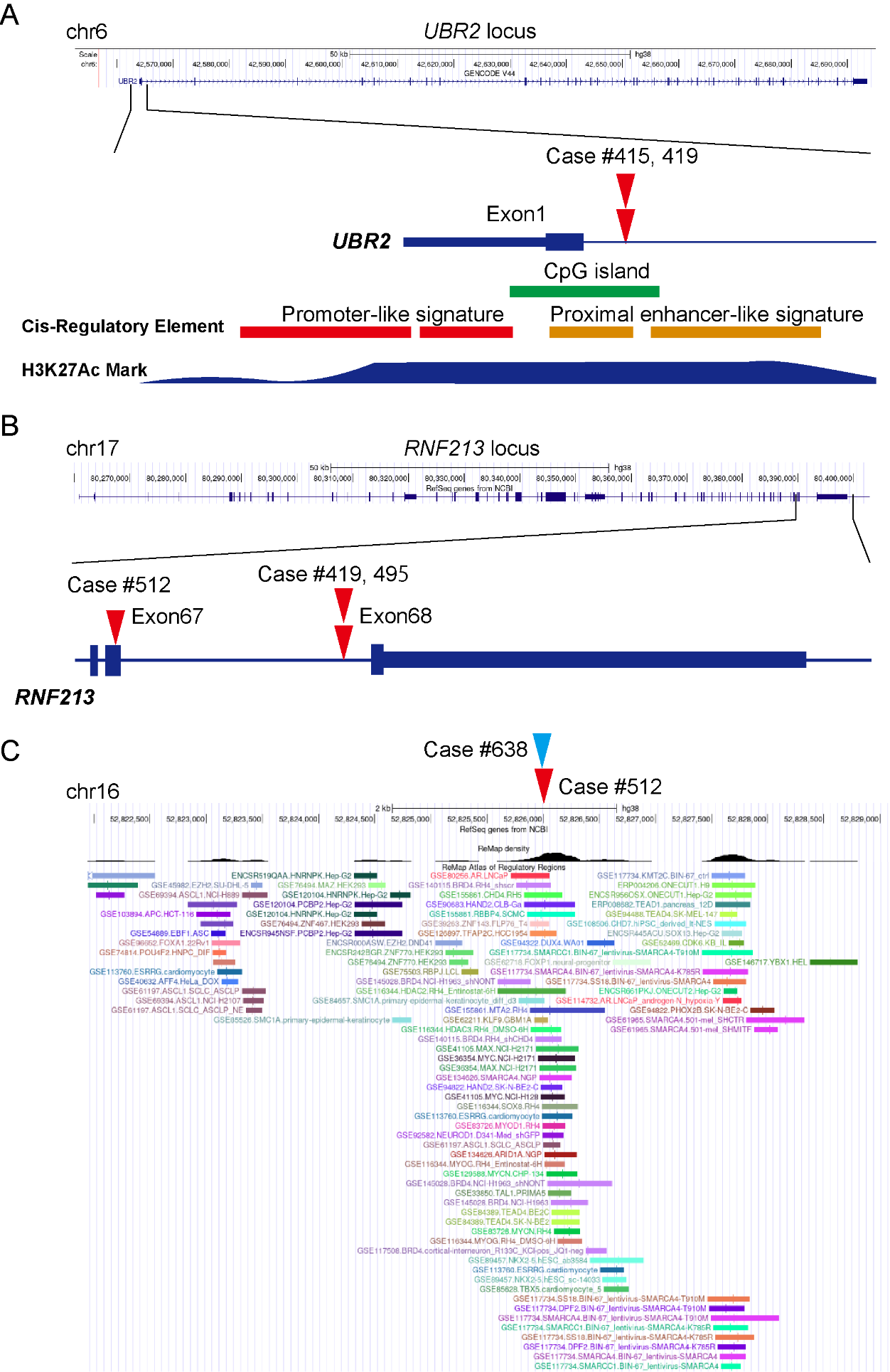


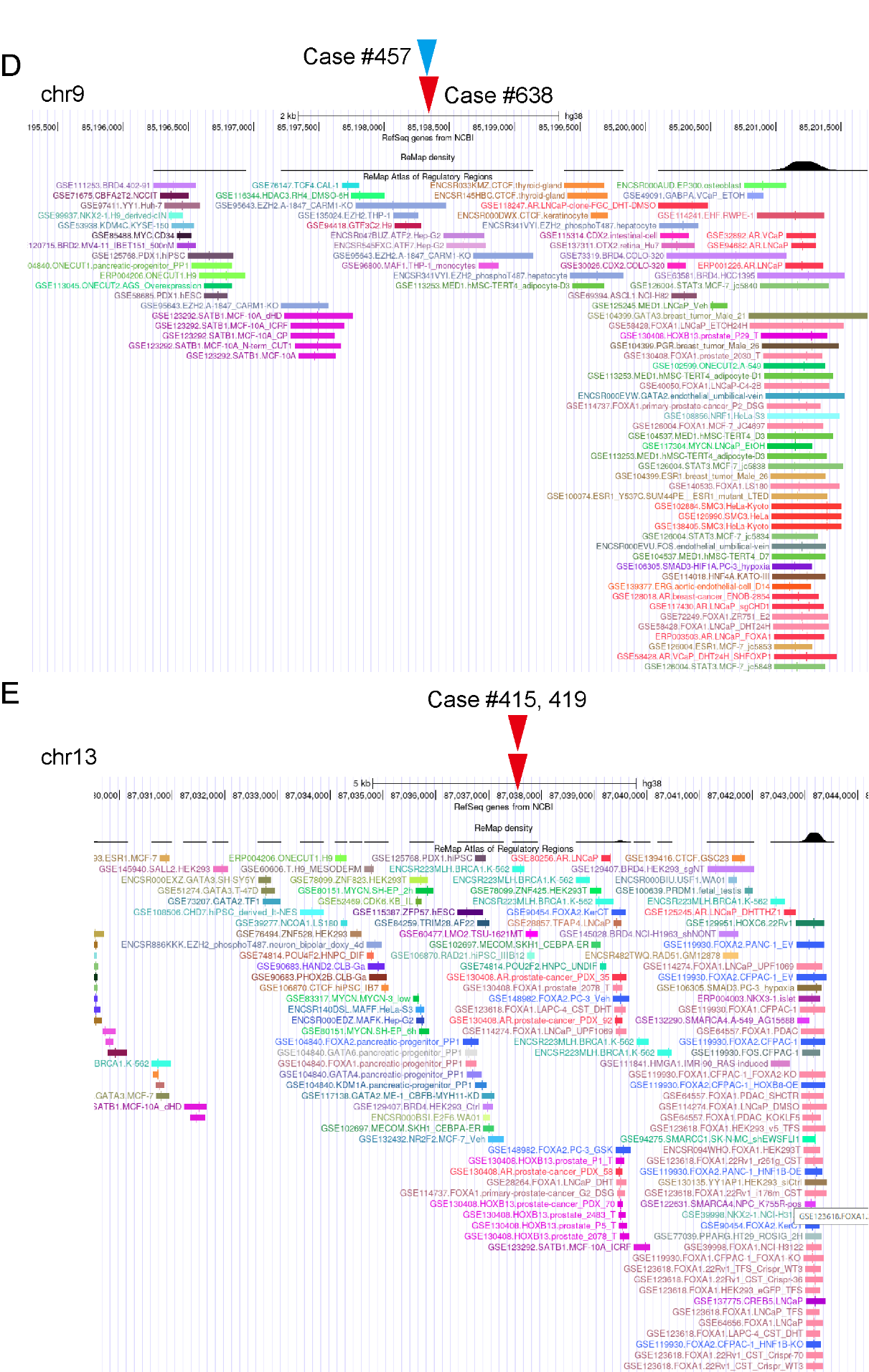


**Figure S5. Recurrent noncoding mutations.**


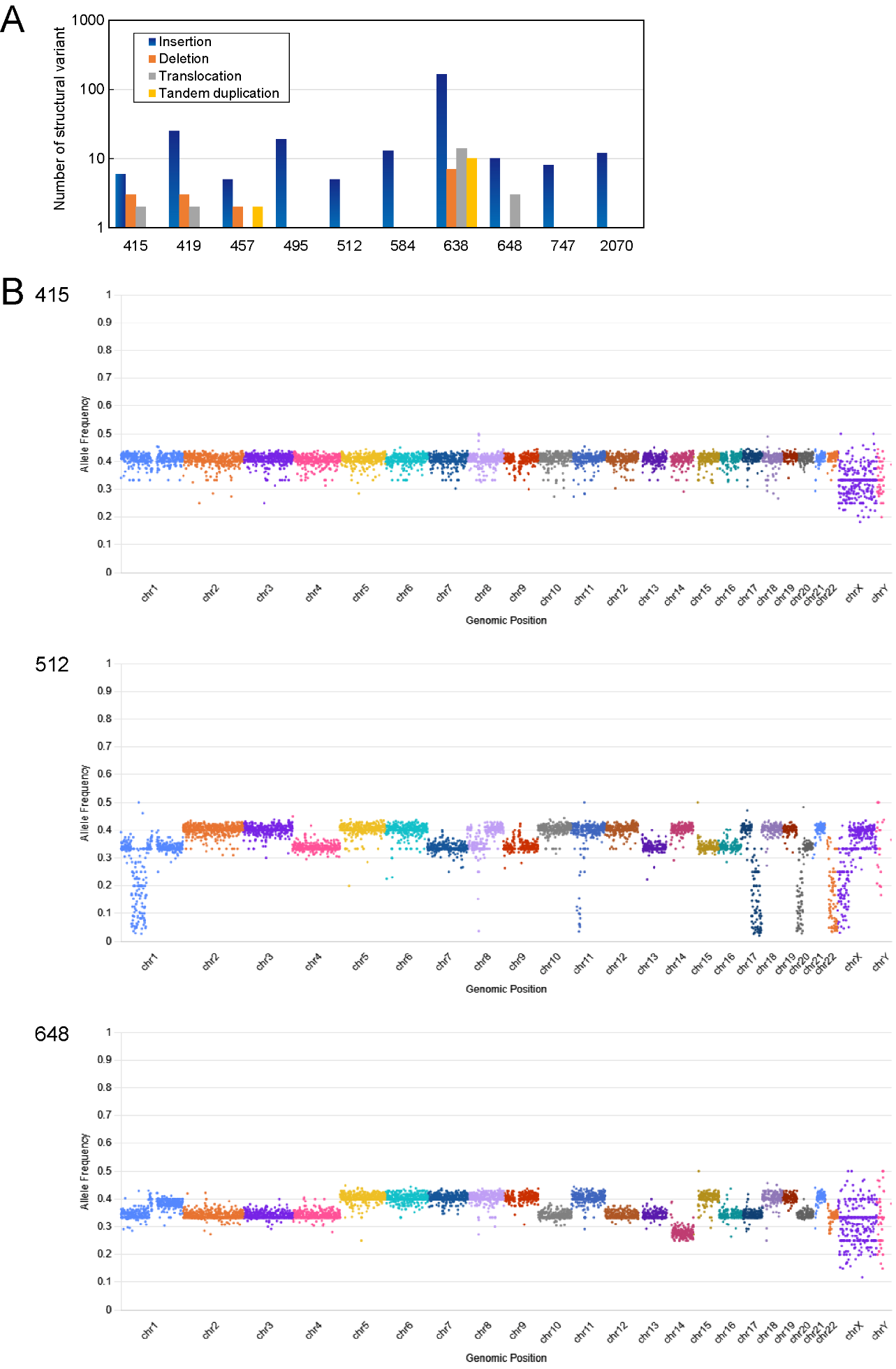


**Figure S6.** **Copy number and structural variants in Type B1 and B2 thymoma.** (A) Number of copy number and structural variants. (B) B-allele frequency for CNVs generated from DRAGEN somatic (v.4.2.7).

**
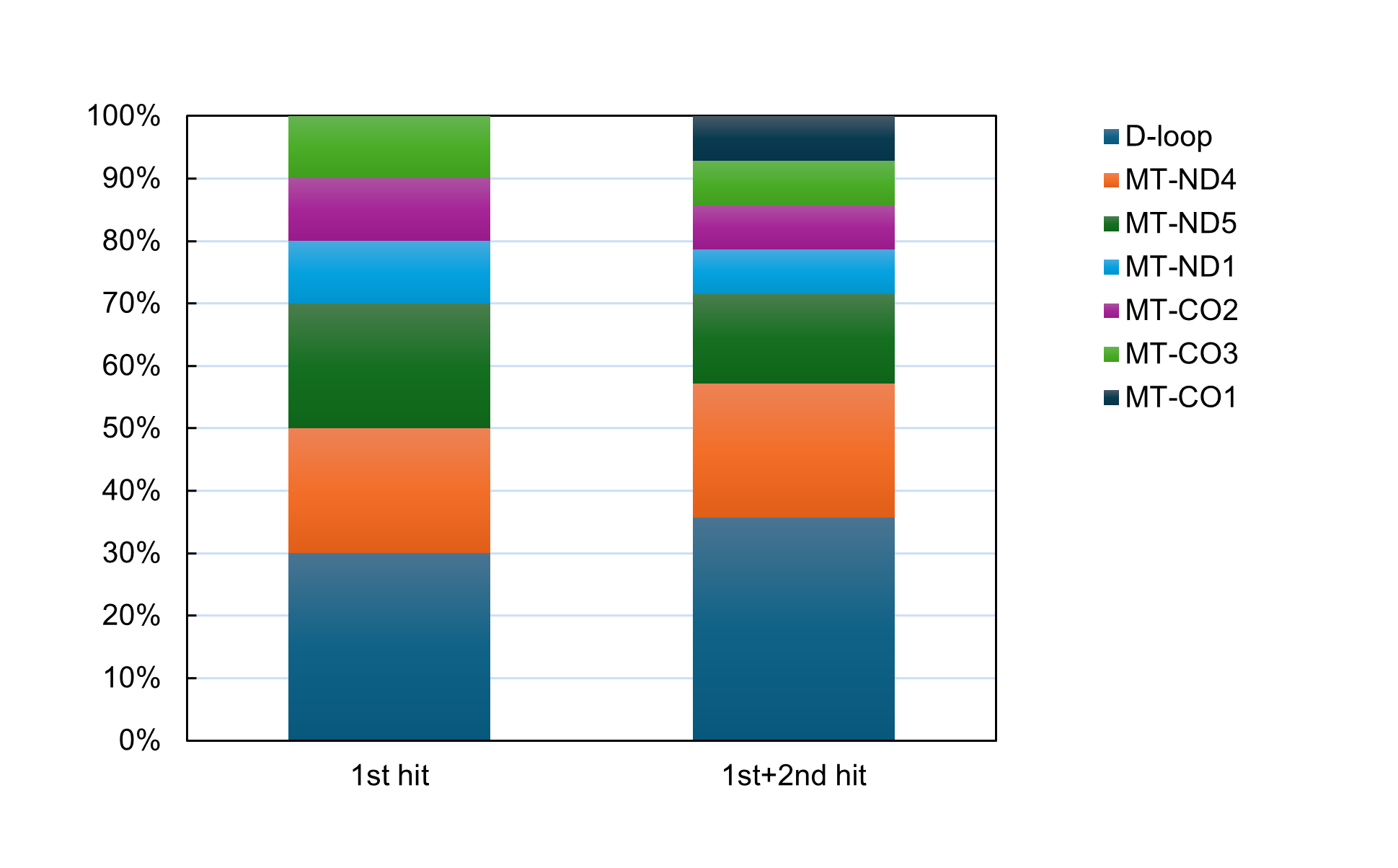
**

**Figure S7. Frequency of primary and secondary hit mutations in the mitochondrial genome.**
